# Stem cells and other innovative intra-articular therapies for osteoarthritis: what does the future hold?

**DOI:** 10.1186/1741-7015-10-44

**Published:** 2012-05-02

**Authors:** Jasvinder A Singh

**Affiliations:** 1 Medicine Service, Birmingham VA Medical Center and Department of Medicine, University of Alabama, Faculty Office Tower 805B, 510 20th Street S, Birmingham, AL 35294, USA; 2 Center for Surgical Medical Acute care Research and Transitions (C-SMART), Birmingham VA Medical Center, Birmingham, AL, USA; 3 Division of Epidemiology, School of Public Health, University of Alabama, Birmingham, AL, USA; 4 Department of Orthopedic Surgery, Mayo Clinic School of Medicine, Rochester, MN, USA

**Keywords:** osteoarthritis, intra-articular, novel, treatment, gene therapy, stem cell

## Abstract

Osteoarthritis (OA), the most common type of arthritis in the world, is associated with suffering due to pain, productivity loss, decreased mobility and quality of life. Systemic therapies available for OA are mostly symptom modifying and have potential gastrointestinal, renal, hepatic, and cardiac side effects. *BMC Musculoskeletal Disorders *recently published a study showing evidence of reparative effects demonstrated by homing of intra-articularly injected autologous bone marrow stem cells in damaged cartilage in an animal model of OA, along with clinical and radiographic benefit. This finding adds to the growing literature showing the potential benefit of intra-articular (IA) bone marrow stem cells. Other emerging potential IA therapies include IL-1 receptor antagonists, conditioned autologous serum, botulinum toxin, and bone morphogenetic protein-7. For each of these therapies, trial data in humans have been published, but more studies are needed to establish that they are safe and effective. Several additional promising new OA treatments are on the horizon, but challenges remain to finding safe and effective local and systemic therapies for OA.

Please see related article: http://www.biomedcentral.com/1471-2474/12/259

## Background

Osteoarthritis (OA) is the most common type of arthritis and the leading cause of disability in the United States [1]. OA alone is responsible for $3.4 to $13.2 billion in job-related costs every year in the US. [2,3] and is associated with significant healthcare utilization, deficits in quality of life, and productivity loss [4-7]. Several systemic treatments, mostly symptom-modifying rather than disease-modifying agents, are available for OA [8]. Recently published OA treatment guidelines highlight the strength of evidence for various therapies [9-12]. However, there is a real need for effective, safe, disease-modifying OA therapies that can not only effectively treat those with established OA, but also possibly delay or prevent progression in those with early OA [13]. None of the potential therapies discussed in this editorial have been approved by regulatory agencies such as the US Food and Drug Administration (FDA), and therefore these therapies are experimental.

## Stem cells for OA: a potential new treatment on the horizon?

Stem cells can differentiate into different cell lineages due to their self-renewing and clonogenic capabilities [14]. Embryonic stem cells have the capability to differentiate into any terminally differentiated cell in the body [15]. Adult stem cells were originally believed to only differentiate into tissue-specific cells. However adult stem cells may be programmed under specific signals to differentiate into other organ-specific cells with a phenotype distinct from that of the precursor. Certain barriers that exist to achieving this effectively *in vivo *must be overcome, namely, easy accessibility to sufficient concentration of stem cells at the site of tissue repair and generation of appropriate signals from the tissue repair site directing the cells to the site [15]. Stem cells can be administered via systemic intravascular route or a direct local implantation, such as that done to repair infracted myocardium [16,17] and in spinal cord injuries [18]. In a recent study by Mokbel *et al. *in *BMC Musculoskeletal Disorders*, labeled autologous adult stem cells suspended in hyaluronic acid were injected intra-articularly into carpal joints in an experimental arthritis induced by intra-articular (IA) Amphotericin-B in donkeys [19]. Significant improvement was noted in clinical and radiographic OA and significantly lesser histopathological changes of OA were seen in carpal joints that received IA autologous mesenchymal stem cells compared to control contralateral joints that received IA hyaluronic acid [19]. Importantly, injected stem cells were incorporated into the articular cartilage of the injected joint, as evident by their integration in the surface of the cartilage and also the interior of the cartilage. Interestingly, while some of these cells showed a chondrocyte-like phenotype indicating their differentiation, other injected cells retained spindle-like structure, characteristic of the mesenchymal origin. Previous studies have suggested that bone marrow and synovial mesenchymal stem cells have more chondrogenic potential compared to adipose or muscle mesenchymal stem cells [20]. While other studies have provided evidence that stem cells may offer potential therapeutic benefit in OA [21,22], challenges remain in the translation of this knowledge into available therapies for patients with OA. The challenges include homing of adequate number of cells in the tissues undergoing repair, long-term safety of such approaches especially those using viral vectors, the durability of the benefit, and feasibility of providing these treatments in busy practitioners' offices. Despite the challenges in bringing this potential therapy to clinic, stem cell therapy offers a revolutionary approach to the treatment of OA.

## New pharmacotherapies for intra-articular use in osteoarthritis

While stem cell therapy may constitute a potential therapy for OA patients in the future, there is need for additional new effective and safe treatment options. Currently available systemic treatments for OA symptoms are commonly associated with gastrointestinal, hepatic, renal, and/or cardiac adverse events, especially in the elderly [8]. This makes IA and local therapies attractive options, especially for patients with limited OA in the knee or hip joints. The counter-argument is that OA is a systemic disease in many patients with involvement of several joints, and therefore there is also a great need for new systemic therapies. Additionally, IA administration may provide a higher concentration of the medication in the joint macro and micro environment, including the cartilage and synovium, and avoid several systemic adverse events [23]. The disadvantage of rare infection following IA injection (0.002%) [24] is far outweighed by its advantages. We discuss a few interesting potential new IA therapies with evidence of early efficacy in human OA. A more complete list is provided in Table 1.

**Table 1:** Emerging intra-articular therapies for osteoarthritis

Therapy/Drug development stage	Proposed mechanism of action
*Evidence from Human Studies*	
Interleukin-1 (IL-1) inhibitor [25,26,44] 2 Phase II/III trials	Inhibition of IL-1, a pro-inflammatory cytokine that promotes cartilage degradation
Human autologous conditioned serum [27,28] 2 Phase II/III trials	Production of anti-inflammatory cytokines such as IL-1 receptor antagonist, IL-4, IL-10, and IL-13
Botulinum toxin [29-32] 3 Phase II/III trials	Inhibition of release of release of Substance P, calcitonin gene-related protein and glutamate from primary afferent neuron terminals
Recombinant human bone morphogenetic protein-7 (BMP-7) [38,45] 1 Phase I trial	Stimulation of proteoglycan, collagen and hyaluronic acid synthesis; induction of receptors, prevention of catabolism of cartilage components by interleukin (IL)-1
Human platelet rich plasma (PRP) [39,40] Case series (*n *= 14)	Stimulation of chondrogenesis, increase in hyaluronic acid production, stabilization of angiogenesis
*Evidence from Animal Models*	
Fibroblast growth factor-18 (FGF-18) [41,42,46]	Stimulation or stabilization of bio-synthesis of cartilage matrix components
Platelet-derived growth factor (PDGF) [43]	Increase in cell proliferation and proteoglycan production; stimulation of meniscal cell proliferation and migration
Insulin-like growth factor (IGF) [42,43,47]	Improvement in cartilage homeostasis by balancing proteoglycan synthesis and breakdown; reduction in synovial inflammation
Caspase inhibitors [48,49]	Inhibition of chondrocyte death apoptosis
Human Kallistatin [50]	Suppression of inflammatory responses and reduce cell apoptosis
Interleukin-6 (IL-6) inhibitor [51]	Inhibition of hypoxia-inducible factor 2 alpha-induced cartilage destruction by regulating matrix metalloproteinase 3 (MMP3) and MMP13
Recombinant human PRG4 (rhPRG4) [52]	Replacement of lubricin, that has lubricant and anti-adhesive properties and is chondroprotective
Recombinant human OPG (rHuOPG) [53]	Suppression of chondrocyte apoptosis
Leptin [54]	Stimulation of synthesis of IGF-1 and TGF beta1 to stimulate chondrocytes to repair extracellular matrix
Mu-opioid receptor [55]	Reduction of substance P and neurotransmitter release from sensory nerve endings peripherally and centrally
Dehydroepiandrosterone [56]	Modulate the imbalance between matrix metalloproteinases (MMPs) and tissue inhibitor of metalloproteinases 1 (TIMP-1)
Berberine, a traditional Chinese medication [57]	Reduction of MMP-3 and MMP-13 levels
*Evidence from in vitro studies*	
Fibroblast growth factor-2 (FGF-2) [58]	Stimulation of chondrocyte progenitor cells
N-acetylcysteine [59]	Increase in chondrocyte viability by reducing oxidative damage

Interluekin-1 beta (IL-1β), thought to play an important role in OA pathology, was targeted with IL-1 receptor antagonist administered IA (at 2 doses, 50 mg and 150 mg) as a single injection in 170 patients [25] and an antibody against IL-1 administered subcutaneously every 4 weeks for three months in 149 patients [26] in one placebo-controlled randomized controlled trial (RCT) each. There were no significant differences in the primary outcome, the Western Ontario McMaster Arthritis Index (WOMAC) scores, at 4-6 weeks follow-up between treatment and placebo groups, in either study. Biochemical and histopathogical changes, including decrease in synovial inflammation and hypertrophy, reduction in highly sensitive C-reactive protein and increase in proteoglycan content, were noted in the patients who received the active treatment. A high placebo response and short half-life of the molecule in the joint may be partially responsible for lack of an effect. It remains to be seen whether modifying the available preparations of IL-1β and/or targeting other cytokine targets in addition to IL-1β, can provide an effective treatment option.

There has been significant recent interest in the use of autologous conditioned serum, which is derived by incubating patient's serum with glass beads to induce the release of several anti-inflammatory cytokines such as IL-1 receptor antagonist, IL-4, IL-10, and IL-13, centrifuged and injected intra-articularly into the joints. However, the early results from two RCTs in humans are contradictory. One RCT of six IA injections showed no significant difference in WOMAC scores (primary outcome) compared to IA saline [27], while the other RCT reported significantly better outcome in IA autologous conditioned serum group, compared to IA hyaluronic acid or IA saline [28].

Another interesting approach is the use of IA botulinum toxin, which is hypothesized to have antinociceptive and possibly anti-inflammatory action. In three RCTs of a single IA injection of botulinum toxin in to 43-60 painful joints each (with painful OA or painful arthroplasty with OA as the underlying condition), clinically and statistically significant improvements in primary outcome of pain as well as extremity function (on WOMAC and shoulder indices) were noted in IA botulinum toxin group (with or without lidocaine) compared to control treatment (saline or saline plus lidocaine) [29-31]. In another RCT, IA botulinum toxin had efficacy similar to IA corticosteroid [32]. Botulinum toxin is known to inhibit substance P and calcitonin-gene related protein [33-36], the main mediators of neurogenic inflammation, a phenomenon of vasodilatation, protein extravasation, and stimulation of inflammatory cells induced by antidromic stimulation of primary afferent fiber [37].

Another therapy on the horizon targets bone morphogenetic protein-7 (BMP-7). In a phase I tolerability and safety study, IA recombinant human BMP-7 showed a higher response rate in treatment compared to the placebo group [38]. Other innovative therapies including human platelet-rich plasma [39,40], fibroblast growth factor-18 (FGF-18) [41,42], platelet-derived growth factor (PDGF) [43], and insulin-like growth factor (IGF) [43] are being tested in early (animal and early human) studies for their potential to repair cartilage (Table 1).

## Conclusions: we are not there yet, but we are on our way

The interesting study by Mokbel *et al. *[18] provides additional evidence from animal models for the potential of autologous mesenchymal bone marrow stem cells as a potential future treatment for OA. Early evidence from human RCTs is also available for additional IA therapies such as IL-1β antagonists, autologous conditioned serum and botulinum toxin. Whether these approaches will translate into effective and safe therapies for humans remains to be seen. This next step will need more convincing evidence of therapeutic efficacy and safety in humans in large RCTs and correlation with improvements in disease pathophysiology, with the use of serum and joint biomarkers and/or imaging biomarkers (radiographs, magnetic resonance imaging, and so on). The future of OA therapeutics seems bright, with a lot of potential therapies targeting different mechanisms of action, different pathways and different approaches for this disabling disease.

## Competing interests

There are no financial conflicts related to this work. JAS has received investigator-initiated research and travel grants from Takeda, Allergan and Savient; consultant fees from Takeda, Ardea, Savient, Allergan, Novartis, and URL pharmaceuticals; and is a member of the executive of OMERACT, an organization that develops outcome measures in rheumatology and receives arms-length funding from 36 companies.

## Authors' contributions

JAS: Concept of the editorial, literature review, preparation of editorial, and revision of the manuscript.

## Author information

JAS is a rheumatologist-epidemiologist, an Associate Professor of Medicine and Epidemiology at the University of Alabama at Birmingham and staff physician at the Birmingham Veterans Affairs Medical Center. JAS is a member of the American College of Rheumatology and serves as a committee member on the Guidelines subcommittee of the American College of Rheumatology's Quality of Care committee, Veterans Affairs Rheumatology Field Advisory Committee, and the executive committee of the Outcome Measures in Rheumatology Trials (OMERACT).

## Pre-publication history

The pre-publication history for this paper can be accessed here:

http://www.biomedcentral.com/1741-7015/10/44/prepub
